# Rebuttal to: Brief behaviour change counselling in the South African context

**DOI:** 10.4102/safp.v67i1.6215

**Published:** 2025-12-19

**Authors:** Olufemi B. Omole, Deidré Pretorius, Klaus B. von Pressentin

**Affiliations:** 1 Division of Family Medicine, Department of Family Medicine and Primary Care, Faculty of Health Sciences, University of the Witwatersrand, Johannesburg, South Africa; 2 Department of Family, Community and Emergency Care, Faculty of Health Sciences, University of Cape Town, Cape Town, South Africa

We thank the authors of the recent letter for their thoughtful engagement with the content of our continuing professional development (CPD) article.^1^ We appreciate the opportunity to clarify and elaborate on several points raised, fostering scholarly dialogue and mutual understanding. This is particularly relevant amid the ongoing theoretical and methodological critiques of motivational interviewing (MI), as discussed by Forsberg, Forsberg and Miller in their recent publication.^2^ During our seminar at the 2024 South African Academy of Family Physicians (SAAFP) Congress,^3^ which laid the groundwork for this CPD article, we presented the 5Rs (relevance, risks, rewards, roadblocks and repetition) approach as a valuable addition to the primary care clinician’s set of strategies for behaviour change in patients who are unwilling or not yet considering change.

Firstly, we recognise that all psychosocial interventions in healthcare (including MI) have developed over time with their own epistemological and ontological assumptions and preoccupations.^4^ It is in this spirit that we aim to contribute to the global discussion on the theoretical and methodological perspectives and principles that underpin behaviour change beyond the MI approach. Davis et al. identified 82 theories of behaviour and behaviour change, drawn from various disciplines such as psychology, sociology, anthropology and economics.^4^ The implementation of MI as a behavioural intervention has been informed not by a single theory but by a multitude of theories. Therefore, it is challenging to distinguish between linear theories of behaviour and more dynamic, cyclical interactional processes in MI, as some approaches may encompass both. Furthermore, these approaches often employ similar constructs – sometimes under different names – and have evolved and merged gradually over time. Although ‘kissing cousins’ do not marry,^5^ their experiences with kissing (along with other factors) repeatedly affect their kissing behaviours in subsequent marriages. In this light, ongoing thorough reviews and reflections offer a fresh understanding of MI within a dynamic context, not limited to linearity. These reflections have also emphasised the lack of theoretical and conceptual stability in the MI approach and suggest that MI may represent ‘general components of good practice’, sometimes called ‘common’ or ‘non-specific’ factors in psychotherapy. Consequently, MI may not be fixed to a single theory nor follow a rigid implementation. Instead, MI embodies knowledge, attitudes and skills that make the helper (the therapist or clinician) more effective for supporting clients in a non-specific and integrative manner.^2^

Secondly, it is essential to consider that the South African primary healthcare landscape (and similar others) is diverse in terms of language, health literacy, ethnicity, culture and beliefs. Our note on MI’s limitations was not a critique, but a reminder that behavioural models often overlook structural health determinants in diverse primary care settings. While MI’s empathy engages social context, our comment about ‘no time limit’ is related to the transtheoretical model (TTM) framework, not MI. We clarify this distinction to avoid misunderstanding. In line with Schouten et al.^6^—and consistent with UNICEF’s social and behaviour change guidance^7^ and LMIC-focused recommendations^8^—we recognise that cultural diversity shapes behaviour-change communication by impacting how messages are crafted, delivered, and received. Differences in communication styles, beliefs, values, and norms can influence intervention effectiveness; accordingly, social and behaviour change communication should be tailored to local cultural and interpersonal contexts to ensure sensitivity and impact, with the required level of specificity varying by setting.^6,7,8^

Briscoe and Aboud argued that no strategy succeeds without all components integrating.^9^ For example, teaching hand washing is pointless if soap and water are not available, regardless of motivation. The diversities of context, culture and clinical services encountered in low- and middle-income countries require flexible, person-centred approaches, not strict algorithms. Context influences patient–doctor interactions and the relevance of frameworks – one size does not fit all. Healthcare variations and patient overload limit the time per patient to 7–10 min, including health promotion and education. In our CPD article, we suggest adding the 5Rs to the existing 5As (assess, advise, agree, assist and arrange) as additional arrows in the healthcare professional’s quiver to navigate difficult conversations, considering system and cultural constraints.^10^

Thirdly, we acknowledge that your letter rightly points out that MI was developed independently of the TTM and is not based on assigning individuals to specific stages. Within the background of TTM, our statement, ‘It posits […]’ in the section on the TTM, referred to the TTM’s description of the stages of change, not that MI allocates people to stages of change. The statement should have read ‘TTM posits that people go through six stages of […]’. Our goal was not to merge the two frameworks but to demonstrate how MI principles can be applied within the context of the four behavioural change models commonly used in clinical practice. However, they are not mutually exclusive; the knowledge of the TTM is an enabler in the implementation of MI, rather than a barrier. An understanding of the TTM, therefore, facilitates discussions about the client’s readiness for change. Our article aimed to reflect this practical integration, rather than suggest a theoretical dependency. Di Clemente summed up the importance of understanding the patient or client’s perspective^11,12^:

The task for individuals in precontemplation is to become conscious of and concerned about the current pattern of behaviour and/or interest in a new behaviour. From a change perspective, it is more important to recognise an individual’s current views on change and address her or his reasons for not wanting to change than it is to understand how the status quo came to be.

Fourthly, we acknowledge the evidence base supporting the locally adapted 5As approach in South Africa and its alignment with MI principles. We welcome the suggestion that the 5Rs are embedded within this adapted 5As approach and agree that integration, rather than dichotomy, is the way forward. We acknowledge that several sources do mention strategies for engaging individuals who are ambivalent or resistant to change. These contributions are valuable and align with our focus on nuanced, patient-centred approaches for individuals who are not yet ready to change. Our article on the 5Rs approach complements these contributions as a complementary practical approach for engaging patients in the precontemplation stage, particularly when traditional behaviour change conversations stall.

In conclusion, we are grateful for the collegial critique and the opportunity to reflect on the different behavioural counselling frameworks. This has enriched our collective understanding, and we hope it will strengthen clinical practice in South African primary care.
